# Regenerative and Immunogenic Characteristics of Cultured Nucleus Pulposus Cells from Human Cervical Intervertebral Discs

**DOI:** 10.1371/journal.pone.0126954

**Published:** 2015-05-19

**Authors:** Stefan Stich, Meaghan Stolk, Pierre Pascal Girod, Claudius Thomé, Michael Sittinger, Jochen Ringe, Martina Seifert, Aldemar Andres Hegewald

**Affiliations:** 1 Tissue Engineering Laboratory and Berlin-Brandenburg Center for Regenerative Therapies, Department of Rheumatology and Clinical Immunology, Charité - Universitätsmedizin Berlin, Berlin, Germany; 2 Institute of Medical Immunology and Berlin-Brandenburg Center for Regenerative Therapies, Charité - Universitätsmedizin Berlin, Berlin, Germany; 3 Department of Neurosurgery, Innsbruck Medical University, Innsbruck, Austria; 4 Department of Neurosurgery, University Medical Center Mannheim, Heidelberg University, Mannheim, Germany; Faculdade de Medicina Dentária, Universidade do Porto, PORTUGAL

## Abstract

Cell-based regenerative approaches have been suggested as primary or adjuvant procedures for the treatment of degenerated intervertebral disc (IVD) diseases. Our aim was to evaluate the regenerative and immunogenic properties of mildly and severely degenerated cervical nucleus pulposus (NP) cells with regard to cell isolation, proliferation and differentiation, as well as to cell surface markers and co-cultures with autologous or allogeneic peripheral blood mononuclear cells (PBMC) including changes in their immunogenic properties after 3-dimensional (3D)-culture. Tissue from the NP compartment of 10 patients with mild or severe grades of IVD degeneration was collected. Cells were isolated, expanded with and without basic fibroblast growth factor and cultured in 3D fibrin/poly (lactic-co-glycolic) acid transplants for 21 days. Real-time reverse-transcription polymerase chain reaction (RT-PCR) showed the expression of characteristic NP markers *ACAN*, *COL1A1* and *COL2A1* in 2D- and 3D-culture with degeneration- and culture-dependent differences. In a 5,6-carboxyfluorescein diacetate N-succinimidyl ester-based proliferation assay, NP cells in monolayer, regardless of their grade of degeneration, did not provoke a significant proliferation response in T cells, natural killer (NK) cells or B cells, not only with donor PBMC, but also with allogeneic PBMC. In conjunction with low inflammatory cytokine expression, analyzed by Cytometric Bead Array and fluorescence-activated cell sorting (FACS), a low immunogenicity can be assumed, facilitating possible therapeutic approaches. In 3D-culture, however, we found elevated immune cell proliferation levels, and there was a general trend to higher responses for NP cells from severely degenerated IVD tissue. This emphasizes the importance of considering the specific immunological alterations when including biomaterials in a therapeutic concept. The overall expression of Fas receptor, found on cultured NP cells, could have disadvantageous implications on their potential therapeutic applications because they could be the targets of cytotoxic T-cell activity acting by Fas ligand-induced apoptosis.

## Introduction

A degenerated intervertebral disc (IVD) is characterized by structural failure together with accelerated or advanced signs of ageing [1], accompanied by inflammatory, catabolic and patho-immunological processes [2,3].

Cell-based regenerative approaches have been suggested as primary or adjuvant procedures for the treatment of degenerated disc diseases [4]. The therapeutic potential of autologous or allogeneic IVD cell transplantation, biomaterials, inhibiting or activating bioactive factors, including gene-therapeutic approaches, have been shown *in vitro*, mostly with animal IVD cells, or in animal IVD models [5,6]. Human degenerative IVD cells, however, were shown to display distinctive features with regard to cell biology and regenerative potential, often differing considerably from animal data [7,8].

Research on regenerative treatment strategies for human degenerative disc diseases has been focused almost exclusively on the treatment of the lumbar spine [9,10]. Beside obvious differences in the biomechanical environment, previous studies have indicated distinct biochemical characteristics of cervical IVDs in comparison to lumbar IVDs [11–13]. Moreover, different pathophysiological processes among various cervical degenerative IVD pathologies might be relevant when designing novel therapeutic approaches [14].

Reviewing the literature, we found only a limited number of regenerative projects reporting specifically on cervical IVD. Luk and colleagues investigated fresh frozen IVD allografting first in rhesus monkeys [15], and then, in five patients with cervical IVD diseases [16]. Despite signs of mild disc degeneration, the motion and stability of the spinal unit was preserved after transplantation and no systemic signs of immune response were observed after five years. Another group approached the cervical IVD in an ovine model with a biodegradable cage, filled with adult allogeneic mesenchymal progenitor cells formulated with a chondrogenic agent [17]. Cartilaginous tissue generation, replacing the IVD following standard anterior cervical discectomy was shown. At a cellular *in vitro* level, cervical NP cells were reported to induce mesenchymal stem cells toward a chondrogenic gene expression profile under co-culture conditions [18]. Moreover, populations of skeletal progenitor cells, capable of chondrogenic differentiation, were found in human cervical degenerated anulus fibrosus and NP tissue [19]. Biological enhancement of cervical degenerated NP cells was shown by gene transfer of the anticatabolic gene *TIMP-1*, resulting in increased proteoglycan synthesis in pellet cultures [20]. Gruber and colleagues contributed several works to cervical anulus fibrosus cells in the context of regenerative approaches, reporting on age as a significant factor for monolayer proliferation [21], on the potential of insulin-like growth factor-1 or platelet-derived growth factor to reduce apoptotic cell death [22], and on successful three-dimensional culture with and without transforming growth factor beta [23,24].

Although many previous findings on regenerative lumbar approaches might also be applicable to the cervical spine; pathophysiological, biomechanical and surgical peculiarities call for a systematic investigation for cervical approaches. Therefore, we examined exclusively cervical disc cells from the NP compartment of patients undergoing surgery for cervical disc herniation via a conventional anterior surgical approach. In this patient collective, mildly as well as severely degenerated IVDs are encountered when graded with magnetic resonance imaging (MRI) [25]. Consequently, these groups were examined separately and then compared in this study.

The main questions we attempt to answer with this study are: (1) can the cervical NP cell isolation and the re-differentiation process after cell expansion be enhanced with basic fibroblast growth factor (basicFGF)? (2) Are there differences between NP cells from mildly and severely degenerated cervical IVDs along the tissue engineering process stages of cell isolation, cell expansion and three-dimensional culture? (3) Do cervical NP cells have the potential to re-differentiate after cell expansion in three-dimensional culture as previously observed with lumbar NP cells [26]? (4) What are the immunogenic properties of NP cells derived from mildly and severely degenerated cervical NP tissue with regard to cell surface markers? (5) How immunogenic are cells isolated from mildly and severely degenerated cervical NP tissues in co-cultures with autologous or allogeneic peripheral blood mononuclear cells (PBMC)? And (6), do the immunogenic properties change after three-dimensional culture in a polymer-based porous biomaterial?

## Material and Methods

### Ethics statement

All subjects participating in this study provided written informed consent to participate in this study, which was approved by the local ethical committee of the Innsbruck Medical University.

### IVD tissue harvesting

**Fig 1:**
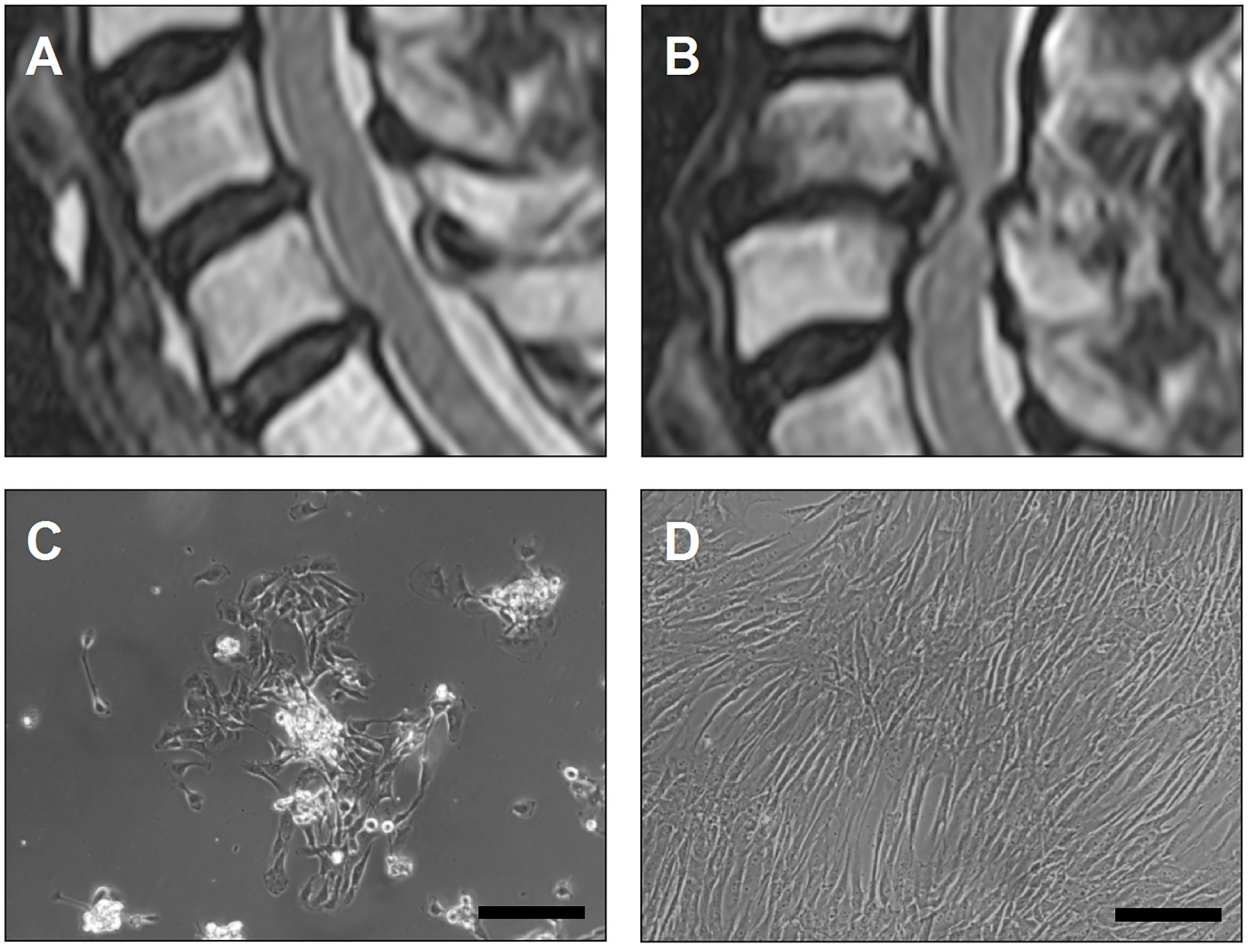
MRT scoring of IVD degeneration and NP cell cultivation. Examples of MRT-scoring for different IVD degeneration grades are given for mild disc degeneration (**A**) and severe disc degeneration (**B**). Cells from all samples were able to proliferate with or without basicFGF, and showed a fibroblast-like morphology (**C**) in primary cell culture. The cells developed a slightly larger, more stretched phenotype (**D**) during cultivation (passage 2); (**C** and **D** 100x, scale bars: 200μm).

Ten patients with cervical disc herniation and symptom duration of a minimum of six weeks were operated via an anterolateral approach, followed by a complete removal of the intervertebral target disc **(** Table 1). Tissue from the nucleus pulposus compartment was carefully selected and transferred to medium consisting of Dulbecco’s Modified Eagle Medium (1 g/mL glucose) (Biochrom, Berlin, Germany) supplemented with 10% human allogeneic serum (German Red Cross, Berlin, Germany), and penicillin and streptomycin (100 U and 100 μg/mL; Biochrom). In order to perform a comparison of mild and severe grades of degeneration, preoperative MRI scans were graded according to the Miyazaki classification [25]. For this study, 5 grade-III discs (donors 1–5, mean age 51 years, standard deviation (SD) 11.7) (Fig 1A) and 5 grade-V discs (donors 6–10, mean age 63 years, SD 10.09) (Fig 1B) were included and defined as mildly and severely degenerated, respectively. Mean age was not significantly different between the two groups (p = 0.119).

**Table 1:** Age, operated cervical segment and corresponding grade of degeneration.

	Age	Segment	Degeneration
Donor 1	54	C5/6	mild
Donor 2	47	C5/6	mild
Donor 3	54	C6/7	mild
Donor 4	34	C5/6	mild
Donor 5	66	C3/4	mild
Donor 6	66	C4/5	severe
Donor 7	73	C5/6	severe
Donor 8	56	C5/6	severe
Donor 9	50	C5/6	severe
Donor 10	72	C5/6	severe

### Isolation and cultivation of NP cells

NP cells were isolated using a modified method as described before [26]. In brief, the tissue was digested with collagenase CLS II (333.3 U/mL) (Biochrom), collagenase P (1 U/mL) (Roche, Mannheim, Germany), and hyaluronidase (33.3 U; Roche) for 2–4 h under continuous stirring in a spinner flask at 37°C and 5% CO_2_. After digestion, the cells were plated with a density of 10^4^ cells/cm^2^. The cells were cultivated at 37°C and 5% CO_2_ with medium supplemented either with or without 2 ng/mL basicFGF (Peprotech, Hamburg, Germany). The medium was changed every 2–3 days. When reaching about 80–90% confluence the cells were passaged using trypsin/ethylenediaminetetraacetic acid (EDTA; Biochrom). For subsequent passages, the seeding density was 5.000 cells/cm^2^. For the tissue engineered grafts, cells were used at the end of passage 2. For investigation of growth kinetics, the NP cells were cultivated up to passage 7 with or without 2 ng/mL basicFGF supplementation with a seeding density of 8.000 cells/cm^2^ at passage 1 and following passages. The volume of medium was 25 mL in each cell culture flask with a surface of 175 cm^2^.

### Surface marker detection

NP cells were labeled with mouse anti-human antibodies to CD54 (intercellular adhesion molecule (ICAM-1) fluorescein isothiocyanate (FITC), CD24 FITC, CD106 (vascular cell adhesion molecule (VCAM-1) phycoerythrin (PE), CD95L (FasL) PE, human leukocyte antigen (HLA)-ABC peridinin chlorophyll (PerCP), and CD95 (Fas) allophycocyanin (APC) (all from Biolegend, San Diego, USA) and HLA-DR APC (BD Biosciences, Heidelberg, Germany) for 30 min and fixed with 1% paraformaldehyde (PFA) (Sigma, Taufkirchen, Germany) until fluorescence-activated cell sorting (FACS) analysis with a BD FACS Canto II (BD Biosciences) and analysis with FlowJo 8.8.5 Software (TreeStar Inc., Ashland, USA).

### Graft construction und 3D cultivation

A non-woven biomaterial that consists of poly(lactic-co-glycolic) acid (PLGA) was used as the basic scaffold. It is biodegradable and the degradation products are non-toxic. The biodegradation starts in aqueous conditions due to nonspecific hydrolytic scission of ester bonds. The degradation product of polyglycolic acid is glycolic acid that will form glycine or can be excreted in urine. Glycine can be used in serine synthesis, or can be converted to pyruvic acid and used in the tricarboxylic acid cycle, ending up as water and carbon dioxide. The degradation product of polylactic acid is lactic acid. This also enters the tricarboxylic acid cycle and eventually becomes water and carbon dioxide. PLGA copolymers have approval from the US Food and Drug Administration and the European Medicine Agency for certain human applications and are used as scaffolds in a variety of orthopedic applications.

For the construction of 3D PLGA/fibrin NP grafts, 2.5 x 10^6^ passage 2 cells were used for each transplant. An amount of 66.7 μl cell suspension in the appropriate cell culture medium with or without basicFGF (Peprotech) was mixed with 33.3 μl fibrinogen (Tissucol Duo S, Baxter, Unterschleissheim, Germany) for one graft. The fibrinogen/cell suspension was soaked into a PLGA scaffold (Ethisorb 5:1, Ethicon, Norderstedt, Germany) with a size of 1 cm x 0.5 cm x 0.2 cm. The scaffold was placed in 30 μl of a 1:10 diluted thrombin solution (Tissucol Duo S, Baxter) and additionally covered with the same amount of it. After a 30 min incubation at 37°C and 5% CO_2_, 4 grafts of the same combination were placed in a cell culture Erlenmeyer flask (Corning, New York, USA) and cultivated together with or without basicFGF supplementation in 15mL medium for 21 days under continuous rotation on a Stovall low profile roller (IBI Scientific, Kapp Court, USA) at 37°C and 5% CO_2_. The medium was changed every 2–3 days.

### Cell survival

To demonstrate cell survival in PLGA/fibrin grafts after 21 days of cultivation, a fluorescein diacetate (FDA; Sigma-Aldrich, Taufkirchen, Germany) staining was performed for 15 min (3 μg/mL) at 37°C to monitor living cells. A propidium iodide (PI) (Sigma-Aldrich) staining was performed for 2 min (0.1 mg/mL) at room temperature to identify dead cells. Microscopic observation of living and dead cells was performed with an Axio Observer fluorescence microscope (Carl Zeiss AG, Jena, Germany) and pictures were taken with an AxioCam MRm (Carl Zeiss AG) camera.

### Histological and immunochemical staining for proteoglycan and collagen type II formation

In order to analyze the production of NP extracellular matrix components in 3D transplants, the grafts were dissected into 4 parts, embedded in TissueTek (Sakura Finetek, Staufen, Germany), frozen in liquid nitrogen and stored at -80°C until sectioning. Cryosections with a thickness of 6 μm were prepared. To demonstrate proteoglycan formation, Safranin O staining was conducted. The samples were incubated for 30 min with a 0.7% Safranin O solution (Sigma-Aldrich) and subsequently counterstained with a 0.2% Fast green solution (Sigma-Aldrich). For the immunochemical detection of collagen type II, a primary monoclonal rabbit-anti-human antibody (Acris Antibodies, Herford, Germany) was used. The detection was performed using the DAKO EnVision Kit (DAKO, Hamburg, Germany) according to the manufacturer’s protocol. In brief, the cryosected transplants were incubated with primary antibody solution for 40 min. The secondary antibody (horseradish peroxidase labeled goat-anti-rabbit antibodies) solution was also applied for 40 min. Finally, the cryosected transplants were incubated with the substrate AEC for 10 min and then counterstained with hematoxylin (DAKO) for 10 minutes. Pictures of both stainings were taken using an Olympus CX41 microscope and an Olympus Colorview camera (Olympus Soft Imaging Solutions GmbH, Hamburg, Germany).

### Gene expression analysis of NP cells in native tissue, monolayer expansion and in 3D transplants

To assess changes in gene expression, real-time reverse-transcription-polymerase chain reaction (RT-PCR) analysis of native NP tissue, expanded monolayer cells (Passage 2, with and without basicFGF) and cultivated transplants (21 days, with and without basicFGF) were performed. Therefore, the ribonucleic acid (RNA) was isolated the following way. For monolayer expanded cells at the end of passage 2, the cells were rinsed with phosphate buffered saline (PBS; Biochrom) and lysed with TRI-Reagent (Sigma-Aldrich). For native tissues (100 μg) and transplants, the samples were transferred to a 4.5 mL cryotube (NUNC, Langenselbold, Germany), frozen in liquid nitrogen and stored at -80°C until cell lysis. Thereby, tissues/transplants were disrupted in TRI-Reagent (Sigma-Aldrich) with an Ultra Turrax (IKA, Staufen, Germany) at maximal speed. After disruption, more TRI-reagent was applied until the liquid was clear. To all lysed monolayer cells, native tissues and transplants, 133μl/mL bromochloropropane (Sigma-Aldrich) of TRI-Reagent was added and shaken for 20 min at room temperature. After centrifugation at maximum speed at 4°C, separation of phases was achieved. The upper phase was collected and mixed with an equal amount of ethanol (70%; Merck, Darmstadt, Germany). The subsequent isolation steps were performed with the Qiagen RNeasy Kit (Qiagen, Hilden, Germany) according to the manufacturer’s protocol. The complementary deoxyribonucleic acid (cDNA) synthesis was accomplished using the iScript cDNA synthesis kit (Biorad, München, Germany).

The real-time RT-PCR was performed with a Mastercycler ep realplex (Eppendorf, Hamburg, Germany) using TaqMan primer and probes for *aggrecan* (Hs00153936_m1), *collagen type I* (Hs01076780_g1) and *collagen type II* (Hs00264051_m1) (all: LifeTechnologies, Carlsbad, USA) gene expression with a temperature profile according to manufacturer’s protocol. The gene expression of all samples is based on a C_t_ value and is given as an absolute copy number calculated over a calibration line [27].

### NP cell co-cultures

NP cells were evaluated for induction of immune responses using a 5,6-carboxyfluorescein diacetate N-succinimidyl ester (CFSE; Life Technologies, Darmstadt, Germany)-based proliferation assay. PBMC from the same donor as the NP cells (referred to within the manuscript as “donor”) or an unrelated healthy volunteer (designated as “allo”) were collected with informed consent into citrate blood collection tubes and frozen in liquid nitrogen until use. On day 0, these PBMC were thawed and then labeled with 2μM 5.6- carboxyfluorescein diacetate succinimidyl ester and added to wells of a flat-bottom 96 well plate (Corning Life Sciences, Amsterdam, The Netherlands) pre-seeded with 3 x 10^4^ NP cells on day -1 for a ratio of 1 NP:10 PBMC in 200μL RPMI 1640 supplemented with 100 units/mL penicillin and 100μg/mL streptomycin (both from Life Technologies) and 10% human male heat-inactivated AB serum (Sigma, Taufkirchen, Germany), filtered through a 0.22μm Stericup filter (Merck Millipore, Billerica, USA). Additional wells were created with a 0.5 cm^2^ piece of NP cell-seeded matrix added to 3 x 10^5^ PBMC. Other wells containing only CFSE-labeled PBMC were treated with 2–5 μg/mL concanavalin A (ConA; Sigma) for a positive proliferation control.

After 5 days of co-culture (37°C; 5% (v/v) CO_2_), supernatants were pooled and frozen at -80°C for further cytokine analysis. Photographs were taken of the cultures using the Zeiss Axis Observer Z1 microscope (Carl Zeiss MicroImaging GmbH, Göttingen, Germany). PBMC were harvested and stained with 3 different multicolor staining FACS panels containing combinations of CD3 PerCP-Cy5.5 (BD Biosciences) and CD8 PE, CD4 APC, CD19 PE, CD25 PE, and CD56 APC antibodies (all from Miltenyi Biotec, Bergisch Gladbach, Germany). The PBMCs which were co-cultured with seeded matrix were labeled with a different staining panel containing CD8 PE and CD4 APC (Miltenyi Biotec), along with CD25 PerCP-Cy5.5, CD56 PE-Cy7, and CD3 APC-Cy7 (Biolegend) and V450 labeled LIVE/DEAD Fixable Dead Cell Stain (Life Technologies, Darmstadt, Germany) in FACS tubes (Micronic, Lelystad, The Netherlands), and fixed with 1% PFA until FACS analysis as above. T cell subpopulations, B cells, and NK cells were gated to determine the extent of their proliferation based on CFSE signal reduction.

### Evaluation of cytokines in proliferation assay supernatants

Supernatants were evaluated for interleukin (IL)-2, IL-4, IL-6, IL-10, tumor necrosis factor alpha (TNFalpha) and interferon gamma (IFNgamma) content using the BD Cytometric Bead Array (CBA) Human Th1/Th2 Cytokine Kit II (BD Biosciences) performed according to the manufacturer’s instructions, measured by FACS and analyzed by FCAP Array v3.0 software (all BD Biosciences).

### Statistics

All data are presented as means ± standard error of the mean (SEM). Data were analyzed for statistical significance by one or two way analysis of variance (ANOVA) with the Bonferroni post-test for differences between groups using GraphPad Prism v5 software. P-values < 0.05 were considered significant (*).

## Results

### Cultivation and cell growth

Cells from all samples (mildly and severely degenerated tissues) were able to proliferate under both conditions (with or without basicFGF). They showed a comparable fibroblast-like morphology (Fig 1C) after isolation and developed a slightly larger, more stretched phenotype during cultivation (Fig 1D). Growth kinetics showed exponential growth curves with mean growth rates (standard deviation) μ_mean_ of 0.189 (±0.035) /day (mild degeneration, + basicFGF), 0.166 (±0.037) /day (mild degeneration,—basicFGF), 0.229 ±0.097) /day (severe degeneration, + basicFGF), and 0.176 (±0.073) /day (severe degeneration,—basicFGF) indicating a higher proliferation of cells from severely degenerated IVD tissue samples. Only cells derived from NP donor 7 showed very slow proliferation (S1 Fig). Cell cultures derived from donors 6 and 8 showed similar growth rates compared to cells from patients with a mild IVD degeneration, whereas cells from donors 9 and 10 developed the highest growth rates measured. The supplementation of basicFGF increased the proliferation in all cell cultures compared to cultures without this factor. Only for donor 1 cells was there a similar growth rate.

### Surface marker expression pattern of NP cells

**Fig 2:**
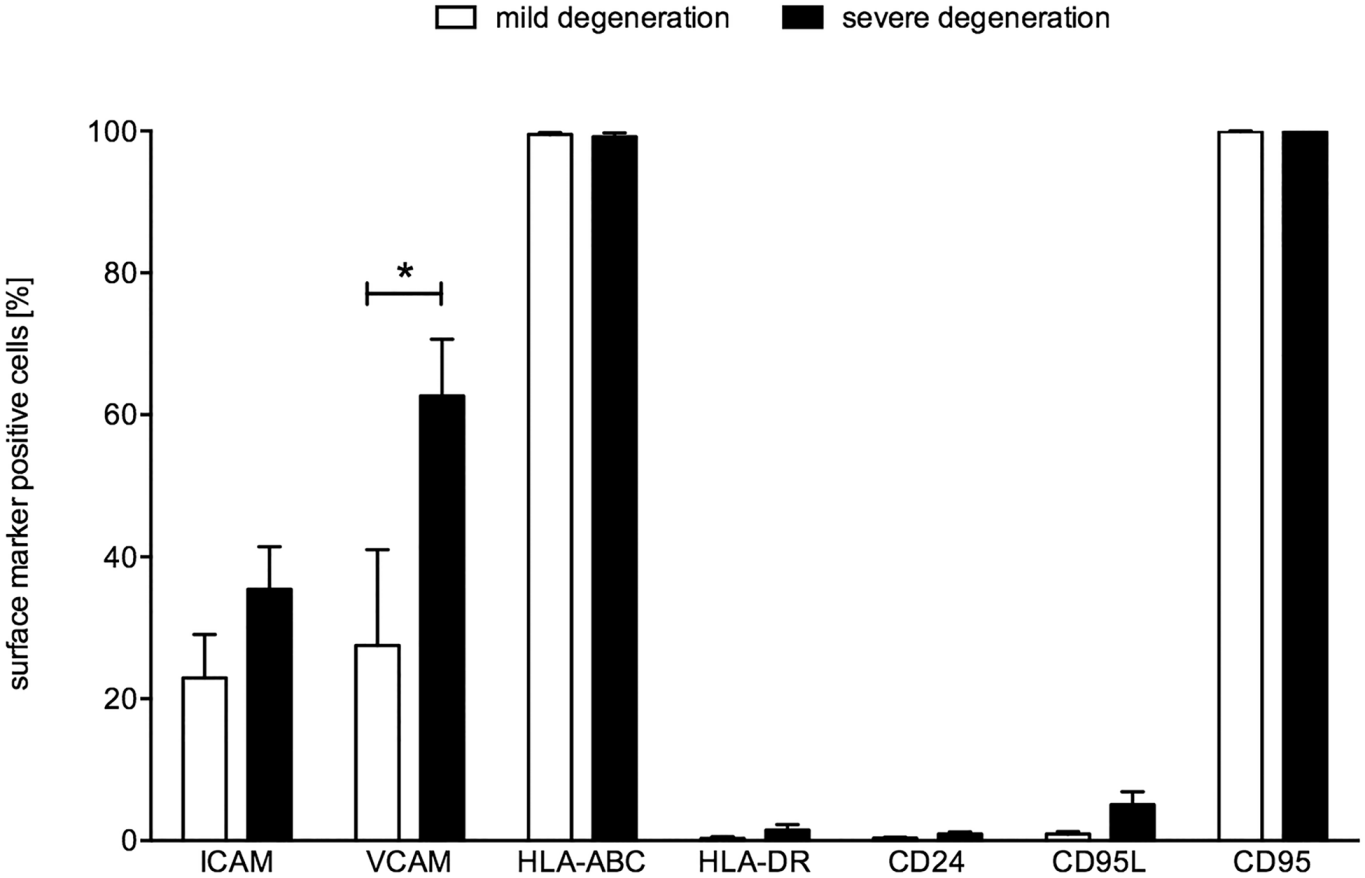
Surface marker expression of NP cells. NP cells were labeled with specific antibodies and analyzed by FACS. The percentage of marker positive cells (mean ± SEM; n = 5) is shown as a comparison between NP cells from patients with mild (white bars) or severe (black bars) degeneration; * p≤0.05.

NP cells at passage 2–3 were tested by flow cytometry for the expression of important surface markers involved in potential interaction with immune cells (Fig 2). Interestingly, the NP cells from donors with severe degeneration tended to have higher expression of the adhesion molecules ICAM-1 and VCAM-1, and Fas ligand, though only VCAM-1 was significantly increased. All cells expressed HLA-ABC and CD95 (Fas), but lacked expression of HLA-DR and CD24.

### Cultivation of 3D NP transplants

**Fig 3:**
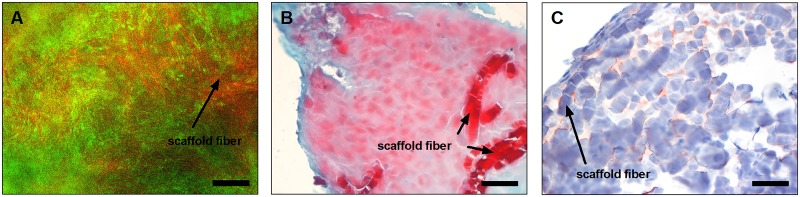
Cell survival and matrix formation of NP transplants. Cell survival of NP cells in transplants cultivated for 21 days was demonstrated for all samples by PI/FDA staining, with living cells appearing green and dead cells in red (**A**). Scaffold fibers appeared slightly red as indicated (see arrow). Proteoglycan production in these transplants was verified by Safranin O staining (**B**) for all samples except for grafts from donor 4. The collagen type II analysis (**C**) revealed only slight staining for transplants of cells from donors 1, 3 and 4 cultivated with basicFGF and for donor 4 without basicFGF; (**A** 100x, scale bar: 200 μm; **B** and **C** 400x, scale bars: 50 μm). Pictures are representative of n = 5 donors.

For each donor, 3 transplants with basicFGF and 4 without FGF supplementation were prepared. The first set was used to demonstrate cell survival after 21 days. The second was used for the evaluation of nucleus pulposus-like matrix formation by histological and immunochemical staining. The third was made for gene expression analysis of typical NP marker genes. The fourth of the samples prepared without basicFGF was used for immunological analysis. After 7 and 21 days, the cell survival was investigated using PI/FDA staining. All cultures showed green stained living cells and only few red stained dead cells independent of the grade of IVD degradation (Fig 3A). There was also no influence of basicFGF on cell survival. No differences were found in transplants cultivated with FGF compared to transplants without supplementation. In order to screen for expressed NP extracellular matrix components, a Safranin O staining was performed to demonstrate proteoglycan formation. All transplants showed positive staining (Fig 3B). Grafts from donors 3 and 8 with FGF and of donor 6 without FGF developed a rather weak formation of proteoglycan. Only transplants of cells from donor 4 demonstrated no proteoglycan content. Furthermore, an immunochemical staining was facilitated to monitor the presence of collagen type II in the transplant. Most of the grafts remained negative. Only transplants of cells from donors 1, 3 and 4 cultivated with basic FGF and from donor 4 without basic FGF revealed slight staining (Fig 3C). The overall scoring of the Safranin O staining and the collagen type II immunochemical staining is given in Table 2.

**Table 2:** Overall scoring of the Safranin O staining and the immunochemical collagen type II staining.

	Safranin O		Collagen Type II	
	+FGF	-FGF	+FGF	-FGF
Donor 1	+	+	(+)	-
Donor 2	+	+	-	-
Donor 3	(+)	+	(+)	-
Donor 4	-	-	(+)	(+)
Donor 5	+	+	-	-
Donor 6	+	(+)	-	-
Donor 7	+	+	-	-
Donor 8	(+)	+	-	-
Donor 9	+	+	-	-
Donor 10	+	+	-	-

stained = +, weakly stained = (+), unstained = -

### Gene expression analysis of NP cells in native tissue, monolayer expansion and in 3D transplants

**Fig 4:**
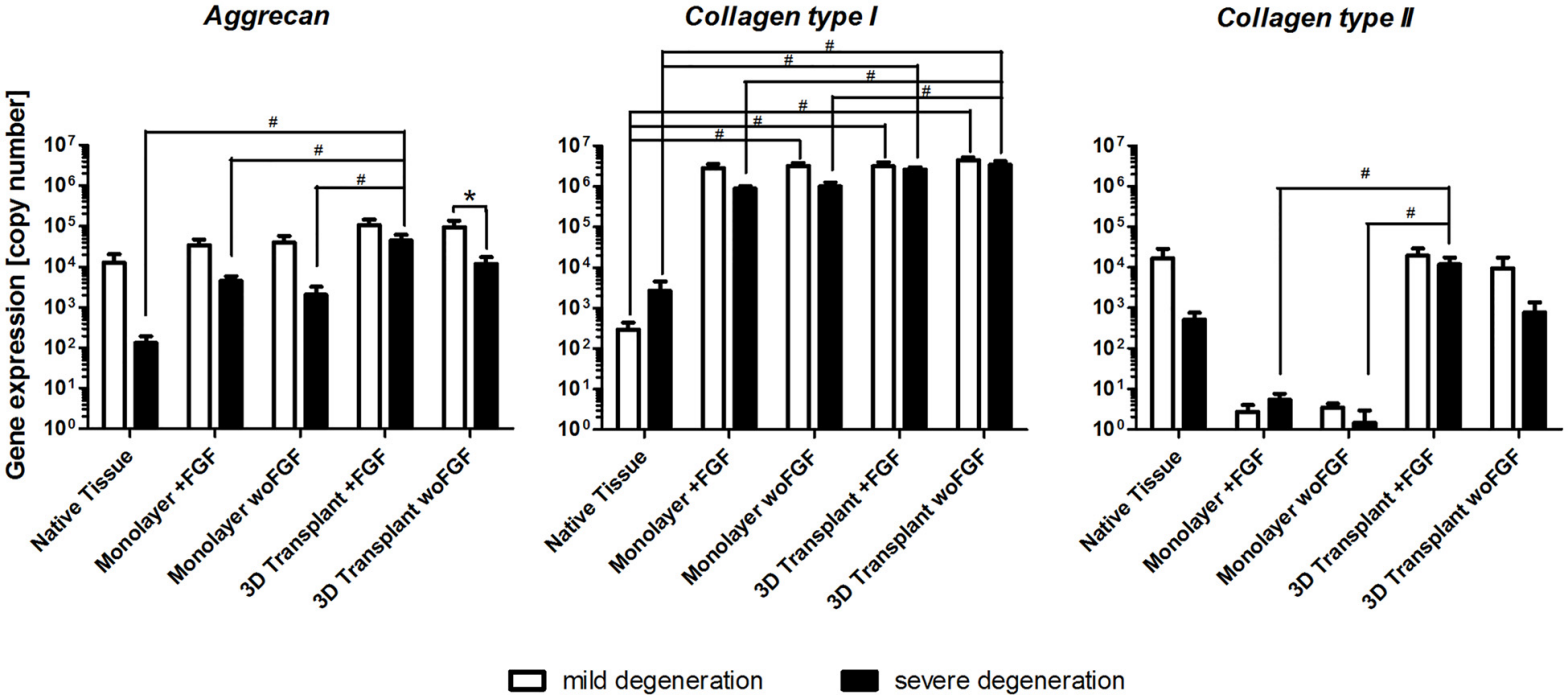
Real-time RT-PCR for NP marker genes. Real-time RT-PCR analysis was performed for the determination of *aggrecan*, *collagen type I* and *collagen type II* expression in native NP, monolayer cultured cells (with or without basicFGF) at the end of passage 2 and NP transplants after 21 days in culture (with or without basicFGF). Results measured as copy number for native NP, monolayer- and 3D-cultures are shown as the mean ± SEM for n = 5 donors; * p≤0.05 between mild and severe groups; # p≤0.05 relative to the donor culture group.

To compare gene expression of common NP extra cellular matrix proteins, real-time RT-PCR was performed *for aggrecan*, *collagen type I* and *collagen type II* using either native NP, monolayer cultured cells (with or without basic FGF) at the end of passage 2 or NP transplants after 21 days in culture (with or without basic FGF). *Aggrecan* expression (Fig 4A) in native NP tissue showed a difference, with cells from mildly degenerated tissues having a higher expression. During monolayer culture the expression was increased and the difference between the degeneration grades diminished. This was further observed in the 3D transplant. Between the cultivation with or without basic FGF, no difference was visible. For *collagen type I* (Fig 4B) the mean gene expression was higher in severely degenerated NP tissues. During monolayer cultivation, the gene expression increased in cells from both degeneration states to a comparable level. This high level in gene expression remained during the 3D cultivation. Again, no difference between FGF treated and non-treated cultures was apparent. For *collagen type II* gene expression (Fig 4C), a higher expression was found in mildly degenerated NP tissue. During monolayer expansion, the *collagen type II* expression decreased to nearly 0. In 3D transplant cultivation, it regained a level similar to native tissue. When comparing different degeneration states in the 3D transplants cultivated without FGF, a higher *collagen type II* gene expression was measured in transplants prepared with cells from donors suffering a mild IVD degeneration.

### Induction of immune cell responses in NP cell and NP cell matrix co-cultures

**Fig 5:**
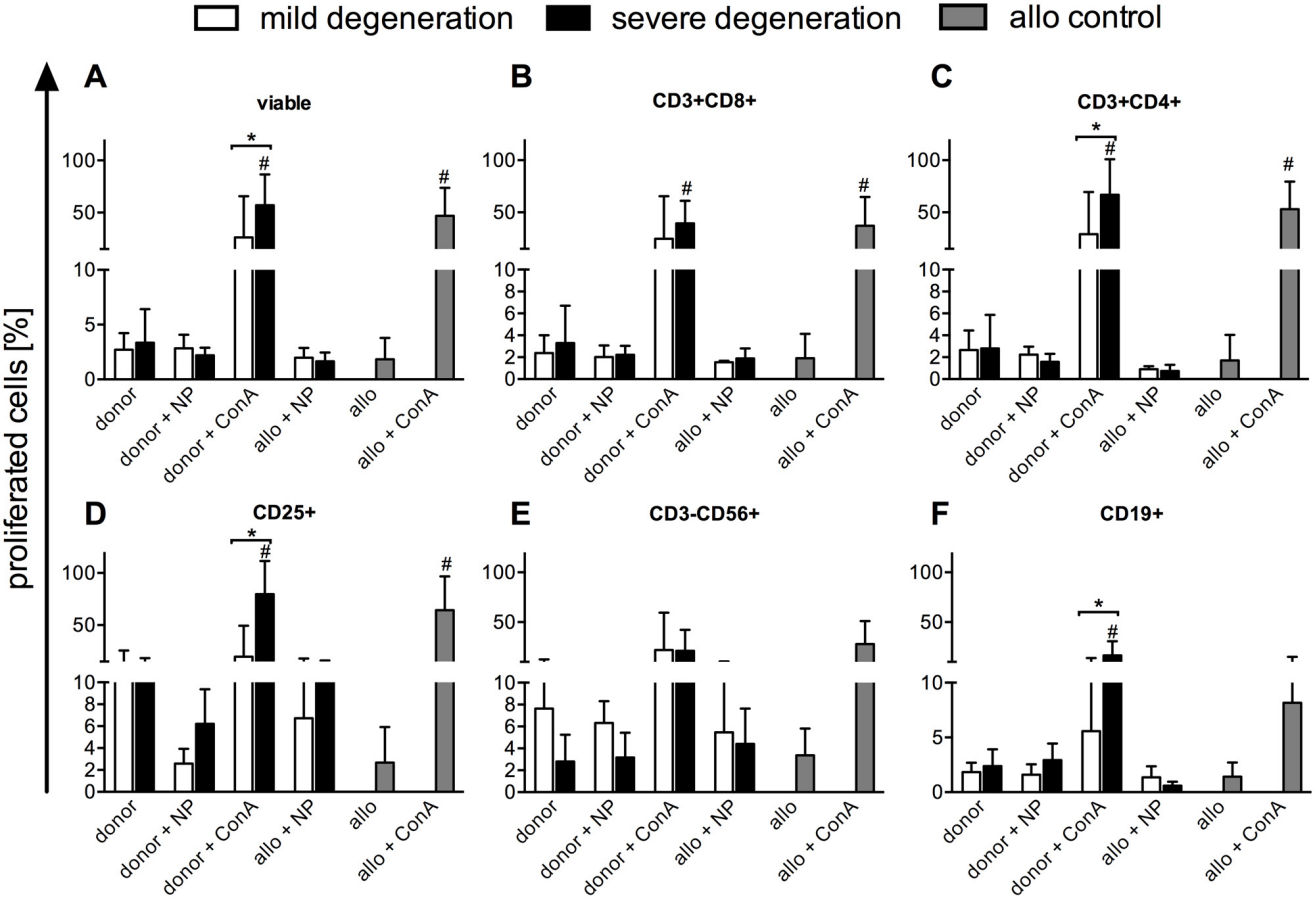
NP cells have low immunogenicity. The immunogenicity of the NP cells from donors with mild or severe degeneration was evaluated using a CFSE-based proliferation assay. PBMC from the same donor as the NP cells (**donor**) or from an unrelated healthy volunteer (**allo**) were co-cultured at a ratio of 1 NP cell: 10 PBMC with pre-seeded NP cells, or stimulated with concanavalin A (ConA) for 5 days. Immune cell subpopulations were identified by gating on antibody-labeled populations and the extent of proliferation was determined from plots of the CFSE signal for: all viable cells **(A)**, the T cell subsets CD3+CD8+ **(B)**, CD3+CD4+ **(C)**, activated CD25+ cells **(D)**, CD3-CD56+ NK cells **(E)** and all CD19+ B cells **(F)**. Results are shown as the mean ± SEM for n = 5 donors; * p≤0.05 between mild and severe groups; # p≤0.05 relative to the donor culture group.

The capacity of NP cells to induce immune cell proliferation of PBMC from either the same donor or an unrelated allogeneic donor was investigated and the summarized data for 5 donors with mild or severe degeneration are shown in Fig 5A–5F. We found that NP cells after monolayer culture did not provoke the proliferation of T cells, NK cells or B cells from either the same donor (the patient which donated the NP cells) or from allogeneic cells (donated by an unrelated third-party). The cells were capable of proliferating however, as proven by their proliferation in the presence of the mitogenic stimulator ConA, with PBMCs from patients with severe degeneration showing higher levels of proliferation for all immune cell subsets. This difference reached statistical significance for all viable cells (Fig 5A), CD3^+^CD4^+^(Fig 5C), CD25^+^ (Fig 5D), and CD19^+^ (Fig 5F), when CFSE-labeled donor PBMCs were stimulated with ConA.

**Fig 6:**
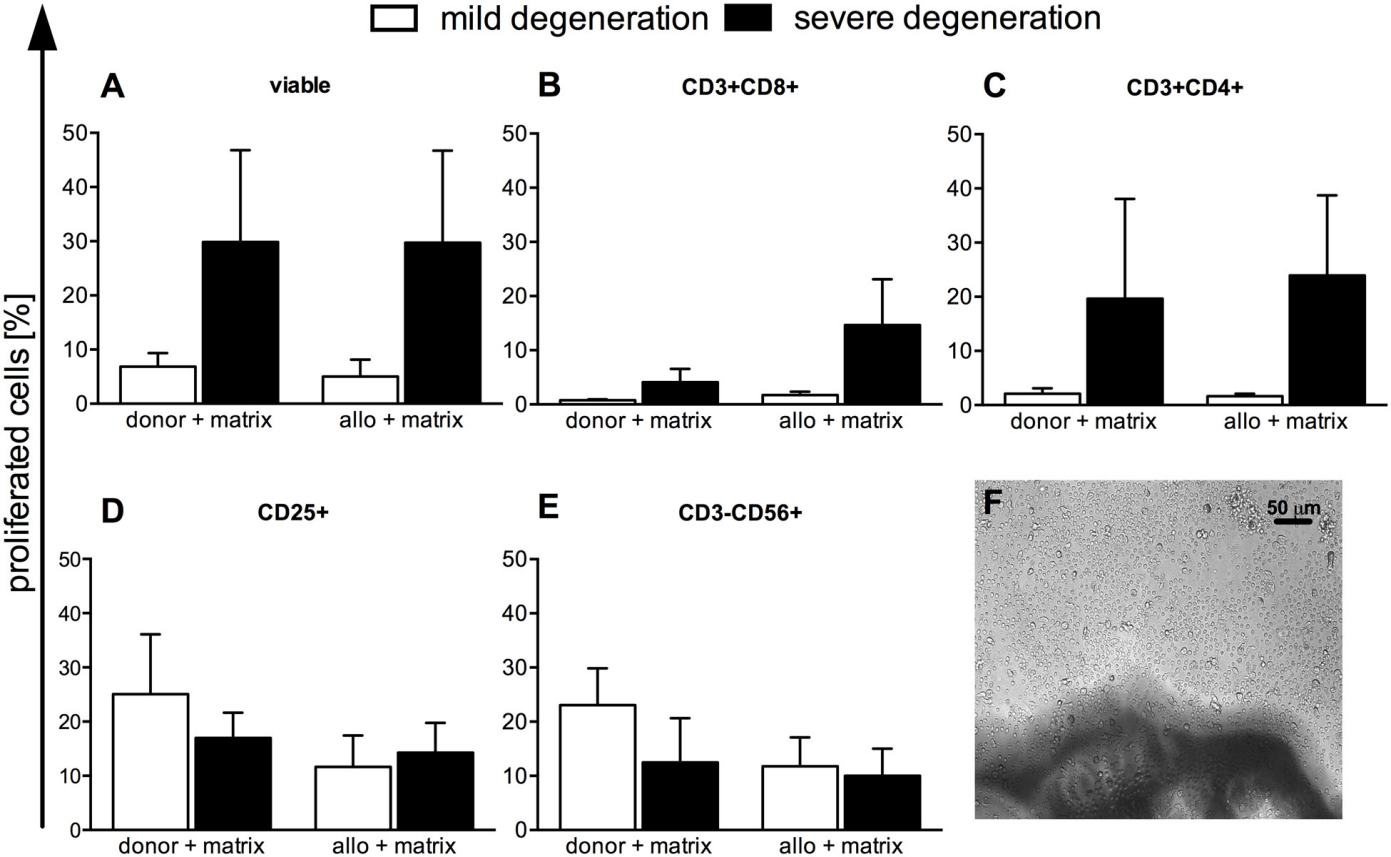
Induction of immune cell proliferation by NP cell seeded matrices. NP cell-seeded matrices were co-cultured with CFSE-labeled donor-type **(donor)** or allogeneic **(allo)** PBMC and incubated for 5 days. Immune cell subpopulations were identified by gating on antibody-labeled populations and the extent of proliferation was determined from plots of the CFSE signal for: all viable cells **(A)**, the T cell subsets CD3+CD8+ **(B)**, CD3+CD4+ **(C)**, activated CD25+ cells **(D)** and CD3-CD56+ NK cells **(E)**. Results are shown as mean ± SEM for n = 5 donors. A representative photograph of matrix seeded with severely degenerated NP co-cultured with allo PBMC is shown **(F)**.

When NP cells were incorporated into a matrix, the immune cell proliferation was provoked to a greater extent than for NP cells pre-seeded as a monolayer when combined with PBMC either from the same donor or from an unrelated allogeneic volunteer (Fig 6A–6E). Matrices containing NP cells from patients with severe degeneration tended to provoke a larger response, especially for CD3+CD8+ (Fig 6B) and CD3+CD4+ (Fig 6C) T cells, than those with mild degeneration, though these differences were not significant.

### Cytokine secretion by immune cells after co-culture with NP cells or NP-seed matrices

**Fig 7:**
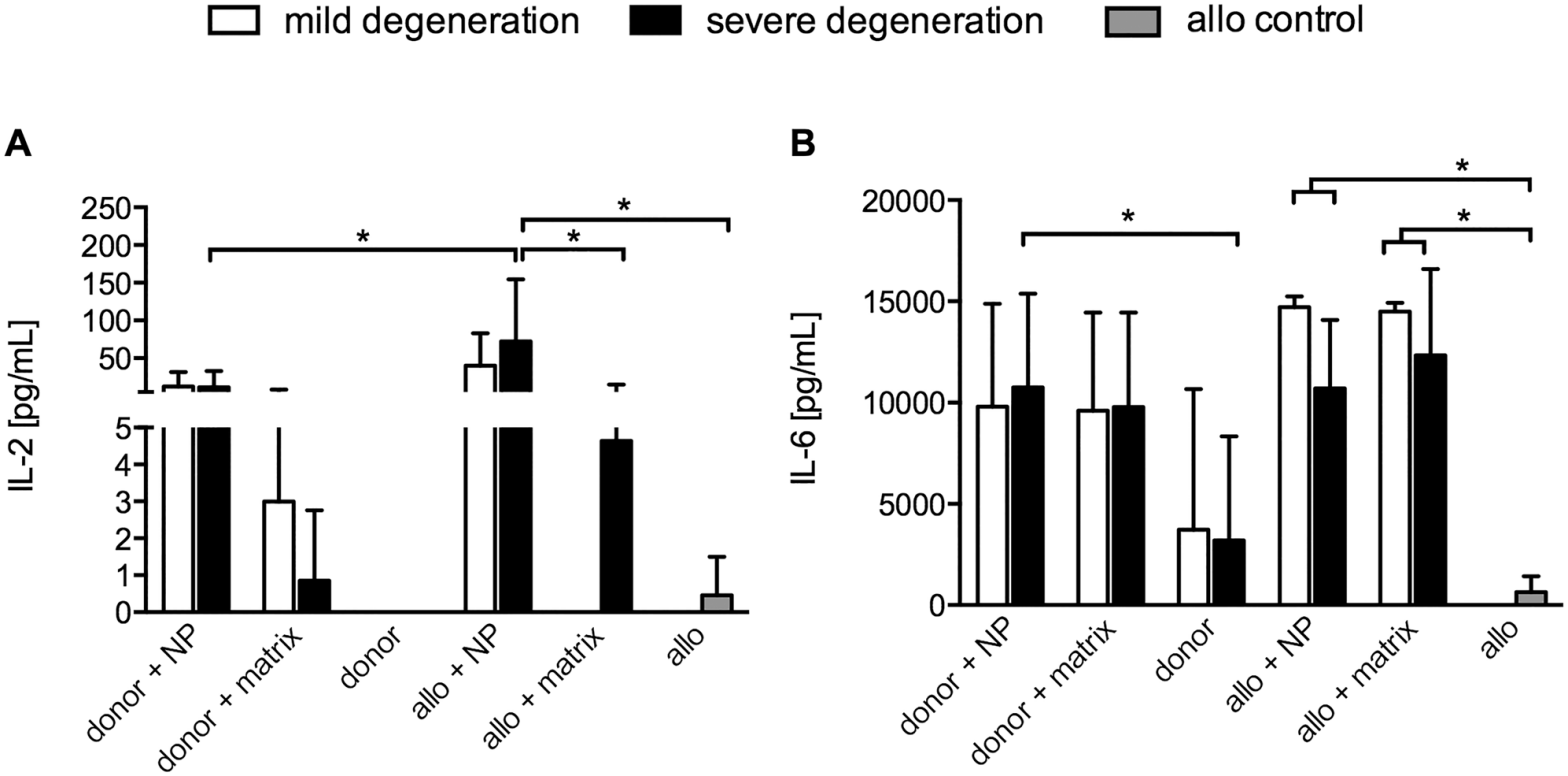
Cytokine secretion in co-cultures of NP cells and NP cell-seeded matrices with immune cells. Supernatants of 5 day co-cultures of PBMC from the same donor as the NP cells **(donor)** or from an unrelated healthy volunteer **(allo)** with NP cells (**donor+NP; allo+NP**) or with NP cell-seeded matrices (**donor+matrix; allo+matrix**) were evaluated for their secretion of IL-2 **(A)** and IL-6 **(B)** by CBA technology. Data are shown as mean ± SEM for n = 5 donors. * p≤0.05 between culture groups. Where no bars are visible, cytokine levels were below the detection limit.

The cytokine release was low or undetectable for the inflammatory cytokines TNFalpha and IFNgamma (data not shown). Similarly, the secretion level of IL-2 was rather low, but tended to be higher in the NP cell co-cultures compared to those with NP cell seeded matrices (Fig 7A). The secretion level was significantly induced in co-cultures of allo PBMC with NP cells of both degeneration grades. Moreover, the IL-2 level was higher in NP cells co-cultured with allo PBMC compared to donor PBMC. Notably, in all co-culture settings with NP cells, the IL-6 release was significantly increased (Fig 7B). Whereas allo PBMC cultures alone secreted 648.3 ± 247.9 pg/mL IL-6, the level increased significantly in co-cultures of allo PBMCs with NP cells of mildly degenerated origin (14710 ± 265.7 pg/mL) or severely degenerated origin (10685 ± 1385 pg/mL). Increased IL-6 levels were also detected in co-cultures of donor PBMCs with NP cells in monolayers, reaching significance for severely degenerated cell sources. NP cells alone were also able to secrete IL-6 (data not shown).

## Discussion

### Cultivation and cell growth

Culture of nucleus pulposus cells has been described as a cumbersome process because of their low cell activity [28]. Previous studies have demonstrated the capacity of FGF to induce proliferation and sustain the potential to produce characteristic matrix molecules of bovine IVD cells [29,30]. Similarly, we previously succeeded in establishing a reproducible lumbar NP cell isolation and proliferation process with FGF [26]. In the present work, FGF increased the proliferation rate in all cervical NP cell cultures, regardless of their origin’s grade of degeneration. Unlike our previous experience with lumbar NP cells, however, sufficient cell proliferation for subsequent three-dimensional culture was also achieved without addition of FGF. Somewhat counterintuitive was the finding that cervical NP cells from severely degenerative IVD showed an increased proliferation rate in comparison to cells from mildly degenerative IVD since ongoing cell death and loss of functionality has often been associated with progressive degeneration [31]. In a recent study, however, no correlation of human IVD cell density to histological grade of degeneration was found [32]. Some previous studies even reported increased cell densities in highly degenerated IVDs [33,34]. Others found that cell cluster formation in human degenerated lumbar IVDs is associated with increased disc cell proliferation [35]. Likewise, gene expression analysis in human degenerated IVDs has shown not only increased expression of catabolic genes, but also some increased anabolic and anti-catabolic gene expressions [36]. In view of these results, one might speculate that the higher proliferation rate found with NP cells from severely degenerated IVDs is due to an ongoing attempt of these cells to counter ongoing degenerative processes in the IVD, whereas NP cells from mildly degenerated discs start off from their normal state of rather low cell and proliferative activity.

Along the tissue engineering process stages of cervical NP cell isolation, cell expansion and three-dimensional culture, gene expression analysis for the typical NP marker genes *aggrecan* and *collagen type II* were performed, along with *collagen type I* as an indicator for a more fibrous de-differentiation. Previous studies with lumbar NP cells in 3D cultures with biomaterials such as polyglycolic acid, alginate or polyurethane, have shown a typical de-differentiation process in monolayer expansion cultures with a decrease in *aggrecan* and *collagen type II* and an increase of *collagen type I* [26,37,38]. However, subsequent re-differentiation in three-dimensional culture towards the original phenotype according to its gene expression profile, was observed in all studies. Two studies noticed a difference in the re-differentiation process depending on the underlying IVD diseases such as disc herniation, osteochondrosis or scoliosis [37,39]. In this study, only tissue from the nucleus compartment of patients with cervical disc herniations was included. As expected, we found higher *aggrecan* and *collagen type II* gene expression rates in native mildly degenerated NP tissue in comparison to severely degenerated tissue. *Collagen type I* was increased in native severely degenerated tissue as previously reported [40]. Contrary to previous results, however, cervical NP cells increased their *aggrecan* expression in monolayer expansion culture, regardless of the grade of degeneration of the original tissue. *Collagen type II*, on the other hand, dropped to almost zero in combination with an increase of *collagen type I* in both groups of degeneration to comparable levels, indicating de-differentiation. Three-dimensional cultivation of the cervical NP cells after cell expansion in a porous polymer-based biomaterial confirmed their potential for re-differentiation with continuous and comparable increases of *aggrecan* expression for both groups of degeneration, as well as increases of *collagen type II* expression to similar levels as in native tissue. Final *collagen type II* expression, however, was significantly higher in the group with mildly degeneration. This was also confirmed with the immunohistological results and remained the only indicator pointing to a functional difference in three-dimensional culture between cervical disc cells from mildly and severely degenerated IVDs. Similar to previous studies, *collagen type I* expression remained high in three-dimensional culture [26,41]. FGF appears to have no significant effect on the re-differentiation process. According to the gene expression results in native tissue, it appears that degenerative changes are predominately due to a loss of NP cell functionality, rather than to a loss of NP cells. Along the tissue engineering process applied in this study, however, these differences appear to diminish.

### Immunogenic NP cell phenotype

For cell-based autologous or allogeneic therapeutic approaches for IVD, it is important to understand the immunogenic properties and induced immune responses of IVD cells. Potential alterations of immunogenic properties along IVD cell isolation and expansion and in three-dimensional culture with various biomaterials have scarcely been addressed in the literature. In this work, we looked at the expression of surface markers relevant for recognition with immune cells and at the induced immune responses of NP cells in co-cultures with peripheral blood mononuclear cells.

The IVD is the largest avascular organ of the body and is suggested to be an immune-privileged compartment [3]. Cells in immune-privileged sites are usually characterized by a low immunogenic surface marker profile.

IVD degeneration leading to structural failures that provide the immune system with access to the immune-privileged compartment, however, can initiate autoimmune processes. This was initially proposed more than 50 years ago [42], and has recently been investigated in several animal studies, which suggested the involvement of early recognition of NP cells by macrophages and NK cells [43], as well as the attraction of T and B cells [44], and the activation of T helper cells by NP cells [45].

In this regard, the Fas ligand / Fas receptor system plays a crucial role. Fas ligand expression is typically found in immune-privileged sites. There, Fas ligand is capable of inducing apoptosis of infiltrating lymphocytes, carrying the Fas receptor. This appears to be a mechanism in the establishment and maintenance of immune-privilege. Fas ligand was shown on human IVDs [46], but appears to display a negative correlation with IVD degeneration in spondylolisthesis [47]. Similarly, another study reported a lower average ratio of positive Fas ligand NP cells in degenerated lumbar IVD without disc herniation (35%), compared to healthy lumbar IVD from cadavers (69%), based on flow cytometry analysis [48]. In human herniated lumbar IVD tissue, Fas ligand and Fas receptor analyzed by immunohistology, correlated significantly with patient age, but not with the degree of disc degeneration on magnetic resonance imaging. Moreover, an increase of Fas ligand (58% to 70%) and Fas receptor (49% to 70%) was found in non-contained disc herniations compared to contained disc herniations, indicating an autoimmunological response to the failure of the natural barrier of the immune-privileged IVD site [49,50]. Similar results were shown in a rabbit IVD stab model and in a rat IVD model [51,52]. In contrast, our FACS analysis demonstrated an incidence of 100% of Fas receptor on isolated and expanded (passage 2–3) cervical nucleus pulposus cells, regardless of their grade of degeneration. Fas ligand on the other hand was comparably rarely expressed with 0.94 ± 0.69% in cells with mild degeneration and showed a trend to slightly higher expression in cells from severe degeneration samples (5.06 ± 4.12%). The overall expression of Fas receptor on cultured cervical NP cells could have a serious impact on their potential therapeutic applications because they would be the targets of cytotoxic T cell activity acting by Fas ligand-induced apoptosis.

We further analyzed the immunological adhesion molecules ICAM-1 and VCAM-1 for their presence on cervical NP cells. In previous studies, ICAM-1 was identified on human lumbar disc herniation tissue. Furthermore, ICAM-1 expression was analyzed after stimulation with IFNgamma, TNFalpha and IL-17 and an up-regulation of ICAM-1 under pro-inflammatory stimulation was reported [53]. To our knowledge there are no data on VCAM-1 on IVD cells. On human articular chondrocytes, however, VCAM-1 appears to play a role in osteoarthritis and rheumatoid arthritis by extravasation of leukocytes from circulating blood to inflamed tissue [54,55]. We found both ICAM-1 and VCAM-1 on cervical NP cells, with a trend toward higher expression of ICAM-1 and significantly higher expression of VCAM-1 on cells from severely degenerated IVDs in comparison with mildly degenerated IVDs. This appears to be in agreement with previous results since progressive IVD degeneration is associated with an inflammatory cytokine milieu that might have triggered the expression of immunological adhesion molecules. We also performed an assay where we stimulated the cervical NP cells for 24 hours with cytokines at 10ng/mL, and found increased ICAM and VCAM-1 expression with IFNgamma, IL-1beta, and especially with TNFalpha (data not shown). In addition, we saw an increase in HLA-DR (major histocompatibility complex (MHC) class II) expression with IFNgamma, which was not expressed at all before stimulation. All of which suggest that a progressive inflammatory environment would affect NP surface markers and could lead to functional changes.

CD24 is another cell adhesion molecule that modulates the B cell activation process. It has been suggested to play a role in NP development and homeostasis [56], and its presence on NP cells was reported to be a differentiating surface marker to anulus fibrosus cells [57]. Both works, however, were based on IVD cells from rats. In the present work, CD24 was not detected on human cervical NP cells.

### Induced immune responses

To characterize the induced immune responses of mildly and severely degenerated cervical NP cells, we performed co-cultures with autologous or allogeneic PBMC containing different immune cell subsets. The cell proliferation and cytokine secretion responses of the PBMCs in co-culture give an indication of the intensity and nature of the immune response. To our knowledge, there are no comparable reports on PBMC co-cultures with NP cells in monolayer or in 3D-culture. We found that human cervical NP cells in monolayer, regardless of their grade of degeneration, did not provoke a significant proliferation response in T cells, NK cells or B cells, not only with the autologous donor PBMC from the same patient which provided the NP cells, but also with allogeneic PBMC from an unrelated donor. Therefore, we found low immunogenicity in this setting. A potential hypo-responsiveness of the allogeneic responder PBMCs could be excluded by measuring a proliferation response within a mixed lymphocyte reaction (MLR, data not shown) or with the mitogen ConA. However, in 3D-culture with a PLGA/fibrin matrix, we found higher immune cell proliferation levels and there was a general trend to more intense responses to NP cells from severely degenerated IVD tissue. We might hypothesize, that higher levels of immune cell responses within the 3D matrix setting are based on so far unknown additional stimuli generated either by new epitope formation or the release of danger associated molecules from the cells or the matrix. Therefore, it is important to consider the immunological implications when introducing specific biomaterials into a therapeutic concept [58].

An unexpected observation was the significantly higher level of proliferation for some immune cell subsets of PBMC from patients with severe IVD degeneration in response to the same dosage of the mitogeneic stimulator ConA that was used to control the general proliferative potential of the PBMC. It might be speculated, that PBMCs of those patients could be pre-activated by inflammatory processes following severe IVD degeneration.

As could be expected from the PBMC proliferation results, overall cytokine induction in PBMC was low. IL-2, a typical T cell cytokine, was released at higher amounts in co-cultures of either donor or allo PBMCs with NP cells or matrices containing NP cells (Fig 7). Elevated IL-2 levels, among many other inflammatory factors, were recently found to be associated with lumbar degenerated disc disease in humans [59]. Surprisingly, other major pro-inflammatory mediators like IFNgamma or TNFalpha were not induced in our assays, with the exception of IL-6, which was significantly increased with regard to the basal level secreted by PBMCs alone after co-culture with NP cells from mildly and severely degenerated discs. We could also show that NP cells of either mild or severe degenerated tissue are able to secrete IL-6. Thus the enhanced level of IL-6 in co-cultures with either donor or allo PBMCs seems to be originating from the NP cells themselves. It could also be possible that NP cells are able to trigger IL-6 release from the PBMCs. Another co-culture study with an immortalized cell line of human NP cells and a macrophage cell line reported a significant increase of pro-inflammatory cytokines compared to the control culture group [28]. Notably, the majority of pro-inflammatory cytokines were produced by the NP cell line. Using the same co-culture system, but with Fas ligand over-expression of the NP cell line, the mRNA and protein expression level of IL-6, but also of IL-1beta and TNFalpha, could be additionally increased. A similar up-regulation of IL-6 in co-culture of rat IVD cells with macrophages was previously reported [60]. Exposure of human NP cells to IL-17 and TNFalpha was able to increase the IL-6 release *in vitro* [53]. The role of IL-6 in the degenerative cascade of the IVD has been studied in several works [12,61–64]. It has been suggested that IL-6 is associated with human IVD degeneration. *In vitro*, IL-6 was observed to up-regulate catabolic gene expression and down-regulate matrix protein gene expression [64].

## Conclusion

In conclusion, cervical NP cells appear to be a suitable source for regenerative treatment approaches with regard to reproducible procedures for NP cell isolation, proliferation and differentiation in 3D-culture. The grade of tissue degeneration and addition of basic fibroblast growth factor had measurable effects on these procedures, but their practical consequences remain unclear. In general, this first attempt for an immunological characterization in this setting demonstrated the low immunogenicity of cultured cervical NP cells, facilitating possible therapeutic autologous and allogeneic approaches. Changes of immunogenic properties after 3D-culture, however, emphasize the importance of considering specific immunological alterations when including biomaterials in a therapeutic concept. At present, our knowledge of immunogenic characteristics and their causes in this context and especially in the in vivo environment that is much different than in cell culture is very limited. Further studies, and defining standardized *in vitro* and *in vivo* strategies for analyzing immunogenic characteristics in new regenerative treatment concepts would be a valuable contribution to make these new technologies available to patients.

## Supporting Information

S1 Fig Cell growth of cultured cells derived from mildly and severely degenerated nucleus pulposus tissue. Cells from all samples (mildly and severely degenerated tissues) could be isolated. They were able to proliferate under both conditions (with or without basicFGF). Compared, cultivation with FGF showed a slightly higher proliferation. Growth curves indicated a slightly higher proliferation of cells from severely degenerated IVD tissue samples. Only cells derived from NP donor 7 showed very slow proliferation. Mean growth rates (standard deviation) μmean of 0.189 (±0.035) /day (mild degeneration, + basicFGF), 0.166 (±0.037) /day (mild degeneration,—basicFGF), 0.229 (±0.097) /day (severe degeneration, + basicFGF), and 0.176 (±0.073) /day (severe degeneration,—basicFGF).
(JPG)
