# Supplementary figures and images for: Regenerative and Immunogenic Characteristics of Cultured Nucleus Pulposus Cells from Human Cervical Intervertebral Discs
Source: PLoS One. 2015 May 19;10(5):e0126954. doi: 10.1371/journal.pone.0126954 (PMC4438063; doi:10.1371/journal.pone.0126954)

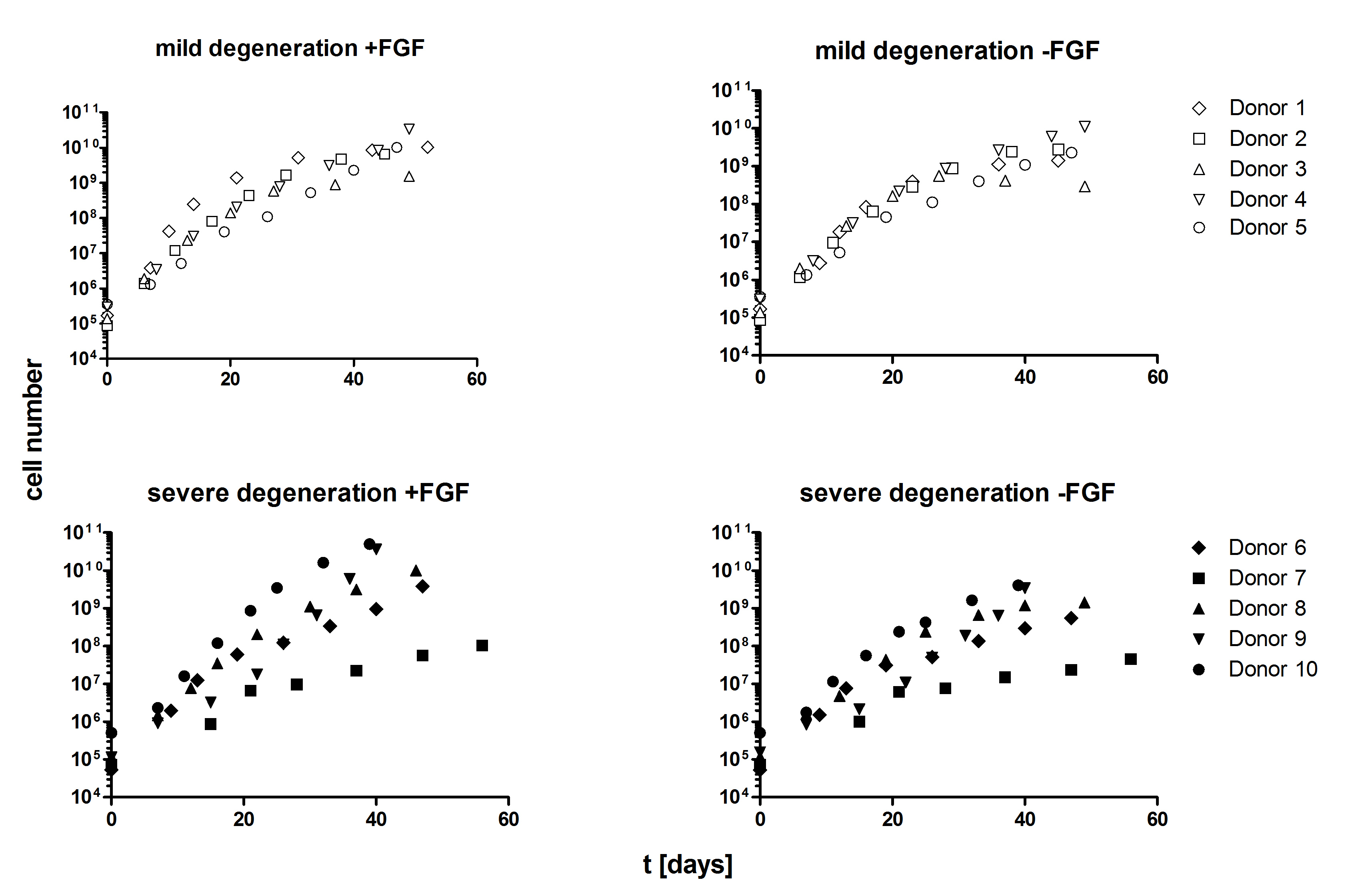

Supplement: S1 Fig — Cells from all samples (mildly and severely degenerated tissues) could be isolated. They were able to proliferate under both conditions (with or without basicFGF). Compared, cultivation with FGF showed a slightly higher proliferation. Growth curves indicated a slightly higher proliferation of cells from severely degenerated IVD tissue samples. Only cells derived from NP donor 7 showed very slow proliferation. Mean growth rates (standard deviation) μmean of 0.189 (±0.035) /day (mild degeneration, + basicFGF), 0.166 (±0.037) /day (mild degeneration,—basicFGF), 0.229 (±0.097) /day (severe degeneration, + basicFGF), and 0.176 (±0.073) /day (severe degeneration,—basicFGF). (JPG) [file pone.0126954.s001.jpg]
